# The Stress-Responding miR-132-3p Shows Evolutionarily Conserved Pathway Interactions

**DOI:** 10.1007/s10571-017-0515-z

**Published:** 2017-06-30

**Authors:** Rotem Haviv, Eden Oz, Hermona Soreq

**Affiliations:** Department of Biological Chemistry, The Silberman Institute of Life Sciences and the Edmond and Lily Safra Center for Brain Sciences, The Hebrew University of Jerusalem, The Edmond J. Safra Campus, 9190401 Jerusalem, Israel

**Keywords:** miRNA-132, Stress, miRNA, Pathway analysis, Cholinergic system

## Abstract

MicroRNAs (miRNAs) are small non-coding RNA chains that can each interact with the 3′-untranslated region of multiple target transcripts in various organisms, humans included. MiRNAs tune entire biological pathways, spanning stress reactions, by regulating the stability and/or translation of their targets. MiRNA genes are often subject to co-evolutionary changes together with their target transcripts, which may be reflected by differences between paralog mouse and primate miRNA/mRNA pairs. However, whether such evolution occurred in stress-related miRNAs remained largely unknown. Here, we report that the stress-induced evolutionarily conserved miR-132-3p, its target transcripts and its regulated pathways provide an intriguing example to exceptionally robust conservation. Mice and human miR-132-3p share six experimentally validated targets and 18 predicted targets with a common miRNA response element. Enrichment analysis and mining in-house and web-available experimental data identified co-regulation by miR-132 in mice and humans of stress-related, inflammatory, metabolic, and neuronal growth pathways. Our findings demonstrate pan-mammalian preservation of miR-132′s neuronal roles, and call for further exploring the corresponding stress-related implications.

**Electronic supplementary material:**

The online version of this article (doi:10.1007/s10571-017-0515-z) contains supplementary material, which is available to authorized users.

## Introduction

MicroRNAs (miRNAs) are short, approximately 20–25 nucleotides long single-stranded RNA molecules that bind to complementary sequences in the 3′-untranslated regions (3′-UTR) of target mRNAs (Lai 2002), referred to as miRNA response elements (MREs). Binding of miRNAs to their targets blocks subsequent protein production by either inhibiting the translation machinery and/or inducing target degradation (Denzler et al. 2014; Meunier et al. 2013), depending on the degree of complementarity (Ambros et al. 2003). Genes encoding miRNAs produce a primary transcript (pri-miRNA), which is co-transcriptionally cleaved by a complex containing the double-stranded RNA-binding protein Pasha and its RNAse counter-part Drosha (Lee et al. 2003; Morlando et al. 2008). The resulting stem-loop pre-miRNA is exported to the cytoplasm by Exportin 5 (Yi et al. 2003), where it is further processed by the endonuclease Dicer into a 20–25 nucleotide double-stranded RNA molecule (Hutvágner et al. 2001; MacRae et al. 2006), altogether leading to massive regulation of the great majority of mammalian genes (Bartel 2009). Individual genes often carry MREs for multiple distinct miRNAs, and conversely, individual miRNAs often target multiple distinct transcripts (Friedman et al. 2009). Genes encoding miRNAs can be transcribed and processed from individual transcription units, from an intron or an exon of a host gene, from an intergenic area, or even from an exon–intron junction of coding or non-coding genes (Nepal et al. 2015; Wanet et al. 2012). Such genes are found in a variety of evolutionarily distant organisms, including vertebrates and plants, and they often show evolutionary differences both in their sequence and targets.

Evolutionary changes in miRNA/target interactions may take different shapes. In both mice and humans, fully conserved miRNAs, such as miR-132, might regulate different targets, or present differential preference for suppressing shared targets, for several reasons: first, alternative transcript variants of a given gene may contain different 3′-untranslated regions (3′-UTR domains), which could either include or exclude MREs (Zhu et al. 2007) or be differently susceptible to miRNA regulation (Mishra et al. 2017). Transcripts undergoing elongation by alternative polyadenylation (Di Giammartino et al. 2011) may likewise present differential target interactions. Additionally, single nucleotide polymorphisms (SNP) in the MRE or close to it could interrupt miRNA binding and/or weaken or strengthen miRNA/target interaction (Hanin et al. 2014; Simchovitz et al. 2017). Also, SNPs located within an adjacent binding site of an RNA-binding protein (RBP) can prevent it from binding; and by doing so, cause a structural modification that blocks a proximal MRE (Kedde et al. 2010).

Both evolution of novel miRNAs and alterations in their target interactions may consist of single nucleotide changes, yet may lead to considerable phenotype differences if not accompanied by parallel adjustment in the target genes. Consistently, primate specification was accompanied by massive coordinated changes in miRNAs and their target genes, leading to primate-specific miRNA populations and indicating potential differences in both their capacity and mechanism of action for controlling specific biological pathways between humans and mice (Barbash et al. 2014); specifically, acute psychological stress responses may be subject to evolutionarily diverse miRNA regulators, reflecting changes in the capacity of species to react to stressful impacts (Hanin et al. 2014; Leung and Sharp 2010; Pandey et al. 2016).

We selected for our current study to focus on miR-132, the stress regulatory capacities of which have been studied for over a decade, especially in the brain, and its many roles were extensively explored using transgenic in vivo models, among other research systems (Edbauer et al. 2010; Jimenez-Mateos et al. 2011; Luikart et al. 2011; Mellios et al. 2011; Nudelman et al. 2010). Those studies showed involvement of miR-132 in neuronal functions, including process extension and neuronal activity. Specifically, transgenic overexpression of miR-132 increases dendritic spine density while causing significant deficits in novel object recognition (Hansen et al. 2010) via suppression of a specific miR-132 target, the p250 GTPase-activating protein (P250GAP) (Wayman et al. 2008); also, miR-132 controls dendritic plasticity by modulating the expression of the stress-sensitive transcription factor methyl CpG-binding protein 2 (MECP2) (Fyffe et al. 2008; Klein et al. 2007) known for its role in the Rett syndrome (Amir et al. 1999). Further, miR-132 is required for the dendritic growth and arborization of newborn neurons in the adult mouse hippocampus (Magill et al. 2010) and regulates structural plasticity of dendritic spines in the mouse through its target matrix metalloproteinase 9 (Mmp9) (Jasińska et al. 2015). Notably, the expression of miR-132 is impaired in Alzheimer’s disease (AD) brains (Lau et al. 2013; Soreq 2015), while Mmp9 levels were shown to be elevated in the plasma of AD patients as compared to controls (Lorenzl et al. 2003).

MiR-132 is also a major regulator of cholinergic signaling (Meerson et al. 2010; Ponomarev et al. 2011; Shaltiel et al. 2013), which is both modulated under stressful insults and subject to complex miRNA regulation (Soreq 2015). The miR-132 predicted binding sites in its target transcripts show consistently lower conservational levels compared to miR-132-3p itself, corresponding to the global phenomenon in which mammalian, and especially primate brain-expressed miRNA genes are evolutionarily more conserved than their predicted binding sites (Barbash et al. 2014). Thus, miR-132 serves as a most appropriate test case for exploring rodent-primate links of the stress-related mode of miRNA functioning.

Regulation of similar targets and/or biological pathways in diverse organisms could potentially indicate control over shared processes by the specific miRNA. Alternatively, or in addition, the miRNA might target other transcripts that belong to the same pathway, and/or compete with other miRNAs on interaction with its targets. To compare the impact of miR-132 regulation in mice and humans, and to explore its implications in stressful situations, we examined both the conservation levels of miR-132-3p targets, and the potential of their interaction with other proteins and the pathways involved in mice and humans.

## Methods

### Identifying the Genomic Location and Structure of miR-132 and Its Predicted Targets

We extracted the mature and pre-miRNA sequences of miR-132 in different organisms from miRBase (Kozomara and Griffiths-Jones 2014), and used the Vertebrate Multiz Alignment & Conservation (100 Species) track in the UCSC genome browser (Kent et al. 2002) and the T-Coffee multiple alignment tool (Notredame et al. 2000) to test for miR-132 conservation. We further assessed its stem-loop structure based on minimum free energy prediction using the Vienna RNAFOLD webserver (Gruber et al. 2008). To find its predicted targets, we uploaded the human miR-132-3p to Diana microT-CDS (Paraskevopoulou et al. 2013; Reczko et al. 2012), with a default threshold of 0.7; and identified those targets that were co-predicted with both Diana microT-CDS and TargetScan (Agarwal et al. 2015).

### Assessing Conserved MREs and Protein–Protein Interactions

We selected experimentally validated MREs using ‘strong evidence’ target validation methods in miRTarBase (Chou et al. 2015). Where we failed to find available validation of an MRE for a certain gene, we employed a predicted general MRE based on the findings in other targets. To test for conservation of MREs between mouse and human, we pursued validated or predicted murine MREs in the human and mouse 3′-untranslated region (3′-UTR) sequences, using the UCSC genome browser (Kent et al. 2002), and limited our selection to predicted targets with fully conserved MREs. Validated and putative miR-132-3p targets in human were then submitted to String (Szklarczyk et al. 2014) with the following parameters: organism—Homo sapiens; prediction methods—Neighborhood, Gene Fusion, Co-occurrence, Co-expression, Experiments, Databases (text mining was excluded); required confidence (score)—highest (0.900). The top 20 interactors that answer those requirements were chosen for further analysis. Network presentation of miR-132, its targets, and their interactors was modified from Cytoscape tool (Shannon et al. 2003).

### miR-132 Overexpression

HEK 293T (ATCC^®^ CRL-3216™) cells were grown in a humidified atmosphere at 37 °C, 5% CO2 in DMEM media supplemented with 10% FBS, 2 mM L-glutamine, 1000 units/ml penicillin, 0.1 mg/ml streptomycin sulfate, and 0.25 µg/ml amphotericin B (Beit-Haemek, Israel). Transfection of 2.5 µg HmiR0268-MR03 plasmid (GeneCopoeia, Rockville, MD) was performed using Polyethylenimine (PEI, SIGMA, St. Louis, MO).

RNA extraction was carried out 24 h post transfection using miRNeasy (Qiagen, Valencia, CA, USA) as per the manufacturer’s instructions. DNase treatment was applied, and RNA concentration and integrity confirmed by Nanodrop and gel electrophoresis, respectively.

RNA samples were used for synthesis of cDNA, using Quanta cDNA synthesis kit for mRNA and qScript microRNA cDNA Synthesis Kit for miRNAs as per the manufacturer’s (Quanta Biosciences, Gaithersberg, MD, USA) protocol. Quantitative real-time PCR (qRT-PCR) was performed on CFX-96 (Bio-Rad, CA, USA) and QuantStudio 12 K Flex-384 (Applied Biosystems, CA, USA) machines using SYBR green master mix (Quanta Biosciences). The mRNA primer sequences are detailed in online Resource 3. Serial dilution of samples was used to evaluate primers efficiency. Long transcript results were normalized to the expression level of RPL19. For miRs, PerfeCTa microRNA assay primers (Quanta Biosciences) were used and results were normalized to the expression of snoRD47. Fold change values for both miRs and mRNAs were calculated using the ΔΔ*C*_t_ method.

### Pathway Analysis

Pathway analysis of enrichment was performed using the David Functional Annotation tool (Huang et al. 2009a, 2009b) with the following parameters: Select identifier: OFFICIAL_GENE_SYMBOL, List Type: Gene List, Background: Homo Sapiens. Both validated and putative miR-132-3p targets in human and mouse, and the proteins they interact with were pursued. The results were retrieved from the KEGG PATHWAY database (Kanehisa and Goto 2000; Kanehisa et al. 2016).

## Results

### Murine and Human miR-132 Genes Share Key Properties

**Fig. 1:**
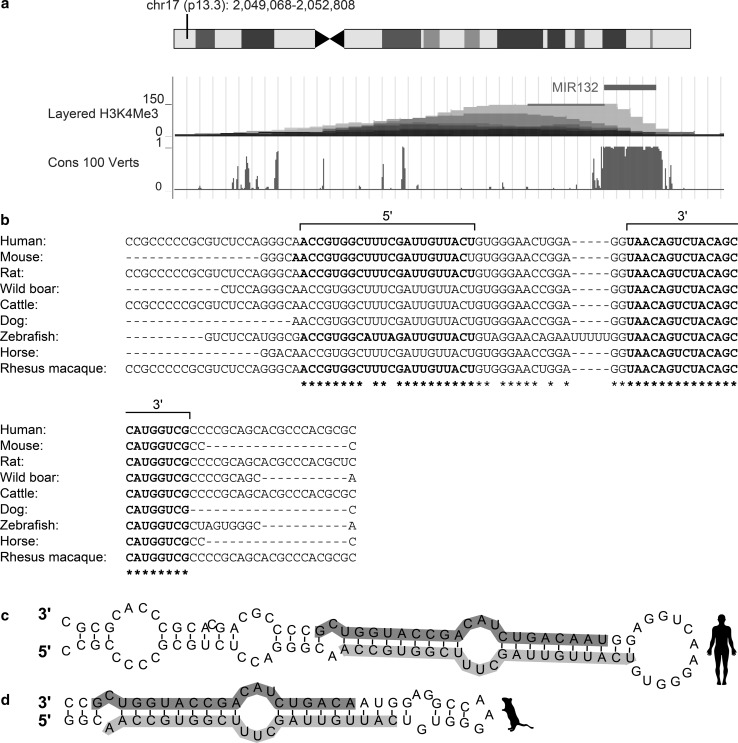
MiR-132 genomic location and structure. **a** Human miR-132′s genomic location, conservation, and promoter-related H3K4Me3 histone modification, adapted from the UCSC genome browser. **b** Stem-loop sequence of miR-132 in different organisms. miR-132 3p and 5p are indicated in bold. **c**, **d** Sequence and predicted stem-loop structure of human (**c**) and mouse (**d**) pre-miR-132. The mature miRNA sequences are indicated in *blue* for the 5′, and *purple* for the 3′. This figure was designed using the Vienna RNAfold webserver based on minimum free energy prediction (Color figure online)

MiR-132 is a highly conserved miRNA that originates from intergenic regions on human chromosome 17 and mouse chromosome 11. Two transcription factors control the miR-132 locus: the cAMP-response element binding protein (CREB), indicating up-regulation under elevated Ca^++^; and the Repressor Element 1 silencing transcription factor/neuron-restrictive silencer factor (REST/NRSF), known to be modulated under aging (Lu et al. 2014) and in AD (González-Castañeda et al. 2013; Lu et al. 2014; Orta-Salazar et al. 2014). Interestingly, one REST and several CREB binding site controllers of miR-132 transcription appear in all mammals, suggesting an evolutionarily conserved involvement of CREB and REST as controllers of miR-132 expression (Remenyi et al. 2010; Wanet et al. 2012; Wei et al. 2013). Furthermore, the genomic site harboring miR-132 displays high conservation levels across vertebrates, mammals, and primates (Wanet et al. 2012). Figure 1a-d presents these shared features for the genomic origin and structure of miR-132 in mice and humans.

### Human and Murine miR-132-3p Share 6 Validated Targets

**Fig. 2:**
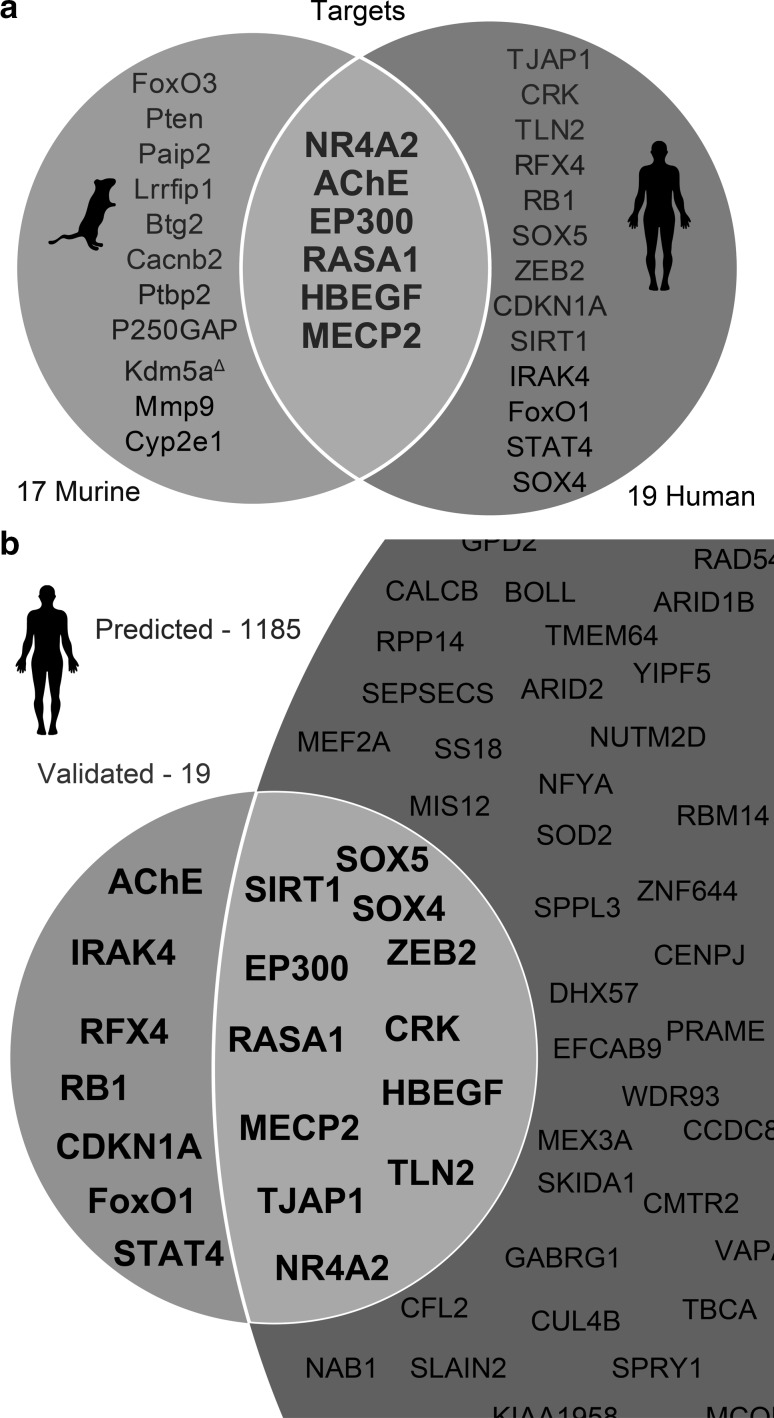
Validated and predicted miR-132-3p targets in mice and humans. **a** Venn diagram of validated miR-132-3p targets in mice and humans. Targets containing validated or predicted conserved MREs are marked in *dark blue* and *dark red*, respectively. Targets without miR-132-3p MRE are marked in *black*. Kdm5a contains only 6/7 nucleotides of the predicted MRE and therefore is marked with (*delta*). **b** Venn diagram of validated and predicted targets of miR-132-3p in human (not to scale). Predicted transcripts were retrieved from Diana microT-CDS using a default threshold of 0.7 (Color figure online)

To gain an insight into potentially shared targets of murine and human miR-132-3p, we searched for its validated human and murine targets in published data and in online bioinformatics tools such as miRTarBase (Chou et al. 2015) and explored the literature for their method of validation. Only targets that were confirmed using high confidence validation methods, such as luciferase assay and western blot, were considered as validated targets. This analysis yielded 19 mRNA transcripts as validated targets of human miR-132-3p (TJAP1, CRK, TLN2, RFX4, RB1, SOX5, ZEB2, CDKN1A, SIRT1, IRAK4, FoxO1, STAT4, SOX4, NR4A2, AChE, EP300, RASA1, HBEGF, and MECP2) (Fig. 2a; Table 1), and 17 as validated targets of murine miR-132-3p (FoxO3, Pten, Paip2, Lrrfip1, Btg2, Cacnb2, Ptbp2, P250GAP, Kdm5a, Mmp9, Cyp2e1, NR4A2, AChE, EP300, RASA1, HBEGF, and MECP2) (Fig. 2a; Table 2), 6 of which were shared between both species. Notably, 5 of those 6 targets (26% of the total validated targets) that were shared between human and murine are stress-related: NR4A2 (Eells et al. 2002), EP300 (Hong et al. 2015), AChE (Kaufer et al. 1998), HBEGF (Zhao et al. 2013), and MECP2 (Fyffe et al. 2008).

**Table 1:** Human miR-132-3p validated targets and the references for their validation

Gene name	References
TJAP1	(Cambronne et al. 2012)
CRK	(Cambronne et al. 2012)
TLN2	(Formosa et al. 2013)
RFX4	(Cheng et al. 2007)
RB1	(Park et al. 2011)
SOX5	(Renjie and Haiqian 2015)
ZEB2	(You et al. 2014)
CDKN1A	(Wu et al. 2010)
SIRT1	(Strum et al. 2009)
IRAK4	(Nahid et al. 2013)
FoxO1	(Li et al. 2015a)
STAT4	(Huang et al. 2011)
SOX4	(Li et al. 2015b)
NR4A2	(Yang et al. 2012)
AChE	(Hanin et al. 2014)
EP300	(Lagos et al. 2010)
RASA1	(Anand et al. 2010)
HBEGF	(Formosa et al. 2013)
MECP2	(Han et al. 2013; Im et al. 2010)

**Table 2:** Murine miR-132-3p validated targets and the references for their validation

Gene name	References
FoxO3	(Wong et al. 2013)
Pten	(Wong et al. 2013)
Paip2	(Alvarez-Saavedra et al. 2011)
Lrrfip1	(Choe et al. 2013)
Btg2	(Alvarez-Saavedra et al. 2011)
Cacnb2	(Carrillo et al. 2011)
Ptbp2	(Smith et al. 2011)
P250GAP	(Vo et al. 2005)
Kdm5a	(Alvarez-Saavedra et al. 2011)
Mmp9	(Jasińska et al. 2015)
Cyp2e1	(Shukla et al. 2013)
NR4A2	(Yang et al. 2012)
AChE	(Shaked et al. 2009)
EP300	(Alvarez-Saavedra et al. 2011)
RASA1	(Anand et al. 2010)
HBEGF	(Molnár et al. 2012)
MECP2	(Alvarez-Saavedra et al. 2011)

A list of predicted targets in human was created through the use of Diana microT-CDS (Paraskevopoulou et al. 2013), cross-checked with the TargetScan (Agarwal et al. 2015) algorithm. Out of the 17 validated targets in murine, 12 were found to be predicted in human. Notably, 37% of the experimentally validated human targets were not predicted by the tools mentioned above (Fig. 2b). Predicting that other targets have not yet been validated, we further searched for transcripts which are validated in murine but not in human; yet share MREs for miR-132-3p in their 3′-compete with each other. We found 6 such transcripts (out of 17), with fully conserved validated MREs in at least one transcript variant of each gene, 3 more carrying a predicted MRE; one of those, Kdm5a, includes a partially conserved MRE (6/7 nucleotides) (Fig. 2a). Those non-validated transcripts were further included in the analysis, as they have a high potential for being viable targets of miR-132-3p, and were hence referred to as “putative targets.”

To further support the expected effect of miR-132-3p on its validated and putative targets, we overexpressed miR-132 in the HEK 293T human cell line and examined selected transcripts for their expression level. We found that the validated target RASA1 and the putative target Paip2 were significantly downregulated upon increase in miR-132-3p levels (*n* = 3, fold change = 0.62, 0.74, 724 respectively; Student’s *t* test: *p* < 0.05). Other validated (SIRT1) and putative (Pten) targets showed non-significant reduction of 20% in their expression levels (*n* = 3).

**Fig. 3:**
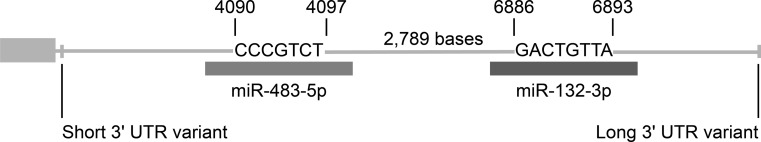
Human MECP2 3′-UTR The MREs for miR-483-5p (*green*) and miR-132-3p (*red*) and their locations in the 3′-UTR of MECP2 are marked (Color figure online)

Predictably, some of the targets do not contain an MRE for miR-132-3p in one or more of their 3′-transcript variants, indicating alternative splicing-dependent regulation. For example, the MECP2 gene contains multiple polyadenylation sites, which result in transcripts with short (approximately 1.8 kb) or long (ca. 10 kb) 3′-UTRs (Klein et al. 2007). We found that the MRE for miR-132-3p is present only in the long variant, which is expressed predominantly in the brain. This finding is supported by experimental evidence, where introduction of miR-132 into primary cortical neurons decreased MECP2 protein levels, while its introduction into L6 muscle cells, which express the shorter MECP2 transcript, did not reduce MECP2 levels (Klein et al. 2007), presenting heterogeneous susceptibility of MECP2 to miR-132 regulation as a result of alternative polyadenylation, similarly to the case of AChE (Mishra et al. 2017). Intriguingly, the MECP2 transcript was further found to be subject to regulation by the human-specific miR-483-5p. In this case (Han et al. 2013), miR-483-5p regulates specifically the long but not the short 3′-UTR variant of MECP2, and a miR-483-5p MRE is found only in the human variant. To examine a possible interaction between miR-132-3p and miR-483-5p regulation on MECP2, we checked the MREs of both miR-132-3p and miR-483-5p in the MECP2 3′-UTR, and found that they are distant enough to ensure that they are unlikely to compete with each other (the 3′-UTR sequence and MREs are presented in Fig. 3).

### MiR-132 Presents a Complex Regulation Network

**Fig. 4:**
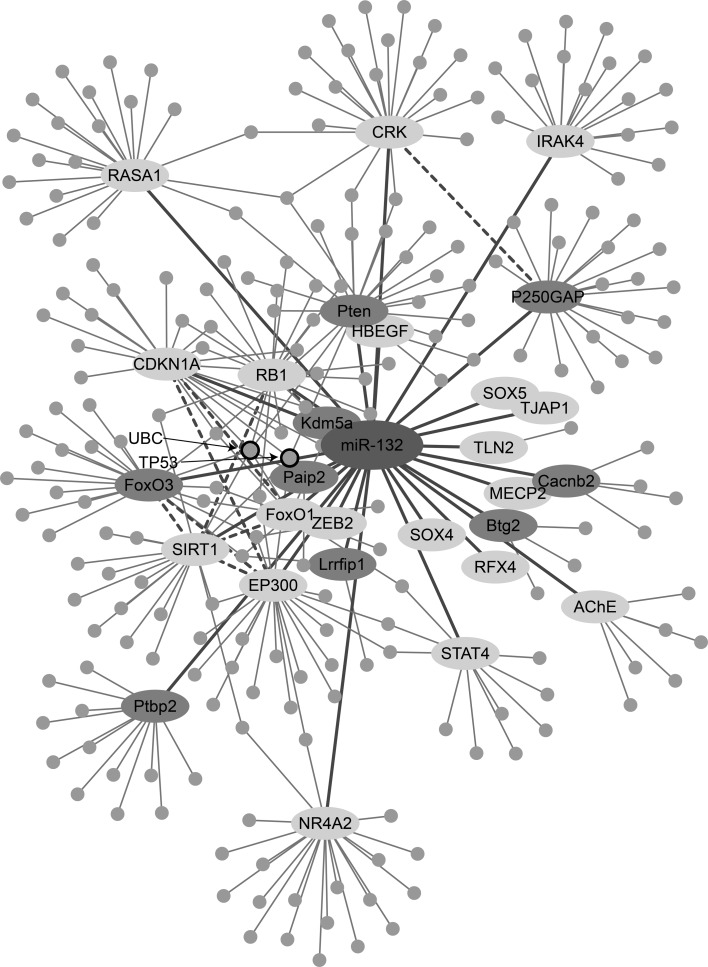
Human miR-132 interaction network. A schematic representation of the interactions between miR-132-3p (*pink*), its validated (*yellow*), or putative (*green*) target genes and the proteins they interact with (*blue*). Interactions between the targets and putative targets themselves are indicated with *dashed-lines* (Color figure online)

To predict miR-132-3p-regulated pathways, we extended our dataset to include proteins which could potentially be affected by miR-132-3p in an indirect manner. For this purpose, we submitted all of the validated and putative targets in human to String (Szklarczyk et al. 2014) and mapped all of the known interactions between them and other proteins (Fig. 4). This analysis revealed a complex pattern of reciprocal interactions between the targets themselves; for example, the EP300 and RB1, validated targets of miR-132, and its FoxO3 putative target emerged as interacting with SIRT1, another validated target (Fig. 4). Further, SIRT1 was found to activate RB1 and bind EP300, which in turn can also activate RB1. A yet more complex interaction was reported between SIRT1 and FoxO3; in the human cell line HEK 293T under oxidative stress, SIRT1 forms a complex with FoxO3 and deacetylates it, potentiating FoxO3′s effect on cell cycle arrest and DNA repair target genes but attenuating FoxO3- dependent apoptosis in the presence of stress stimuli (Brunet et al. 2004). Moreover, mammalian cells which undergo acute nutritional stress present a FoxO3-dependent increase in SIRT1 levels, mediated by an interaction with TP53. Notably, FoxO3-TP53 interaction exacerbates after nutrient withdrawal (Nemoto et al. 2004). Moreover, some proteins, such as Tumor Protein P53 (TP53) and Ubiquitin C (UBC), interact with more than one target of miR-132-3p (Fig. 4). TP53 can induce growth arrest or apoptosis after cellular stresses (Nagashima et al. 2001); it binds SIRT1, EP300, PTEN, FoxO3, and CDKN1A, and is activated by SIRT1 and EP300. UBC, a stress-inducible gene (Figueiredo-Pereira et al. 1997; Tsirigotis et al. 2001), binds the miR-132-3p targets SIRT1, FoxO3, PTEN, PAIP2, RB1, and CDKN1A. Taken together, these findings could indicate miR-132 involvement in several fundamental processes in the cell by affecting key proteins, such as TP53 and UBC. These interactions further suggest that miR-132-3p targets may be regulated both in a direct and indirect fashion, and that this regulation could be stress-affected. Therefore, the question arose if these complex interactions as well are evolutionarily conserved.

### MiR-132-3p is Predicted to Affect Shared Pathways in Mice and Humans

We examined the pathways affected by miR-132-3p both in human and mouse through its targets and the proteins they interact with. Pathway analysis of 247 transcripts in human and 248 transcripts in mouse (including only validated and putative targets, and their interactors) was performed using the DAVID functional annotation tool (Huang et al. 2009a; Huang et al. 2009b). We started by examining the pathways analysis in human; predictably, a large number of the identified transcripts emerged as being involved in several cancer types, such as Prostate cancer, Pancreatic cancer, Glioma, and Melanoma (Online Resource 1, FDR = 6.4E−35, 7.4E−24, 2.2E−22, 8.8E−20, respectively). In addition, intriguing non-cancerous pathways were found to be enriched with genes of interest (Table 3), and some of those pathways showed experimentally validated relation to miR-132 in mouse. A prominent example is the immune system, demonstrated by the predicted involvement of miR-132-3p in T cell and B cell receptor signaling pathways, chemokine signaling pathway, Leukocyte transendothelial migration, and natural killer cell-mediated cytotoxicity (FDR = 4.8E−14, 5.4E−09, 5.2E−12, 5.6E−06, 3.7E−04, respectively), which is compatible with the potentiation by miR-132 of the cholinergic blockade of inflammation (Mishra et al. 2017; Shaked et al. 2009).

**Table 3:** Selected pathways predicted to be affected by miR-132-3p in Human

Pathway	Number of genes	% of involved genes	FDR
Nervous system			
Neurotrophin signaling pathway	32	12.96	7.7E−19
Cholinergic synapse	17	6.88	9.6E−05
Immune system			
T cell receptor signaling pathway	26	10.53	4.8E−14
Chemokine signaling pathway	31	12.55	5.2E−12
B cell receptor signaling pathway	18	7.29	5.4E−09
Leukocyte transendothelial migration	19	7.69	5.6E−06
Natural killer cell-mediated cytotoxicity	17	6.88	3.7E−04
Metabolism			
Inflammatory bowel disease (IBD)	11	4.45	1.4E−02
Insulin signaling pathway	24	9.72	5.3E−09
Insulin resistance	18	7.29	9.5E−06
Type II diabetes mellitus	12	4.86	8.5E−05
Cell cycle			
Cell cycle	38	15.38	1.7E−25
Apoptosis	18	7.29	8.0E−10

Pathways with more than 4% involved genes are listed. For each selected pathway, the numbers (out of 247 genes), percentage of involved genes, and FDR are shown

Yet more specifically, our analysis re-confirmed miR-132′s involvement in cholinergic synapses (FDR = 9.6E−05). The role of miR-132 in the cholinergic system was demonstrated in numerous studies (Meerson et al. 2010; Ponomarev et al. 2011; Shaked et al. 2009; Shaltiel et al. 2013), compatible with its regulatory effect on synaptic transmission (Remenyi et al. 2013). The link between the cholinergic signaling and stress is well profound (Gilad et al. 1985; Kaufer et al. 1998); ACh levels are transiently elevated in the mammalian brain during stress responses (Masuda et al. 2004). AChE, a shared validated target of miR-132-3p in human and mouse, is responsible, together with the homologous enzyme butyrylcholinesterase (BChE), for terminating cholinergic signaling by rapid hydrolysis of ACh in the synaptic cleft (Soreq 2015). In a mouse model of psychological stress, miR-132 was elevated in the hippocampus, accompanied by and associated with reduced AChE activity, which predictably potentiates ACh signaling, and exacerbates anxiety (Meshorer and Soreq 2006; Shaltiel et al. 2013; Soreq 2015). Inversely, mice treated with anti-miR-132 oligonucleotide showed elevated activity of the synaptic variant AChE-S, which may suppress the stress-characteristic hyper-activation of synaptic neurotransmission (Mishra et al. 2017).

Other stress-relevant processes include the Neurotrophin signaling pathway; the neurotrophin growth factors are important in neuronal development and survival as well as in synapse formation and plasticity. One subtype of neurotrophin, BDNF (brain-derived neurotrophic factor), increases miR-132 expression upon its administration to cultured primary cortical mouse neurons (Remenyi et al. 2010). We found that 32 of our tested genes were enriched in the neurotrophin signaling pathway (FDR = 7.7E−19), indicating a potential involvement of miR-132 in both murine and human neurotrophin signaling. Likewise, 16 of the tested genes in the GnRH (Gonadotropin-Releasing Hormone) signaling pathway were found to be related to miR-132-3p regulation (Online Resource 1, FDR = 3.9E−05). GnRH is known to induce the expression of miR-132 in mouse pituitary gonadotroph cells. Subsequently, it reduces the expression of P250GAP (a validated target in mouse), resulting in changes in cellular morphology and increased migration (Godoy et al. 2011). Thus, both direct and secondary processes may jointly lead to miR-132-3p network interactions.

Lastly, miRNAs were suggested to mediate the connection between anxiety and metabolic disorders (Meydan et al. 2016). Compatible with the overlapping elevation of miR-132 in anxiety and metabolic impairments, our enrichment analysis revealed several metabolic disorders, such as non-alcoholic fatty liver disease (NAFLD, FDR = 2.8E−04), inflammatory bowel disease (IBD, FDR = 1.4E−02), and Type II diabetes mellitus (FDR = 8.5E−05), alongside with metabolic-related pathways, such as the Insulin signaling pathway (FDR = 5.3E−09). Validating the functional relevance of this interaction, we have recently shown that mouse models of hepatic steatosis or non-alcoholic steatohepatitis (NASH) display dramatic increases in liver miR-132 levels and corresponding reduction in selected miR-132 targets, whereas antisense oligonucleotide-mediated miR-132 silencing increases the levels of its targets and consequently reduces the steatotic phenotype (Hanin et al. 2017). Further, human patients with inflammatory bowel disease exhibit increased levels of miR-132-3p in intestinal tissue biopsies, with corresponding decreases in circulatory AChE activity, relative to healthy controls (Maharshak et al. 2013), suggesting miR-132 involvement in IBD alongside with its stress-related target AChE. Interestingly, comparing the enriched pathways in human and mouse (Table 4, Online Resource 2) demonstrated that although only half of the identified genes of interest in mouse and half of those in human are shared, the pathways predicted to be affected by them are highly similar; out of 85 pathways in human and 88 in mouse, 74 pathways are shared. Notably, among the pathways that were found to be unique to human is IBD. The metabolic and stress links of miR-132-3p thus point at multiple ailments as stress-associated.

**Table 4:** Selected common and unique enriched pathways in human and mice

Pathway	FDR human	FDR mouse
Cell cycle	1.7E−25	2.0E−19
Neurotrophin signaling pathway	7.7E−19	6.1E−11
T cell receptor signaling pathway	4.8E−14	1.5E−12
Chemokine signaling pathway	5.2E−12	1.9E−11
Apoptosis	8.0E−10	4.0E−06
Insulin signaling pathway	5.3E−09	2.5E−05
B cell receptor signaling pathway	5.4E−09	1.7E−15
Leukocyte transendothelial migration	5.6E−06	4.9E−12
Insulin resistance	9.5E−06	4.6E−07
Type II diabetes mellitus	8.5E−05	4.7E−06
Cholinergic synapse	9.6E−05	1.0E−08
Non-alcoholic fatty liver disease (NAFLD)	2.8E−04	1.7E−02
Natural killer cell-mediated cytotoxicity	3.7E−04	2.1E−07
Shigellosis	1.4E−02	–
Epithelial cell signaling in Helicobacter pylori infection	2.1E−02	–
NOD-like receptor signaling pathway	2.6E−02	–
NF-kappa B signaling pathway	4.0E−02	–
Herpes simplex infection	5.2E−05	–
Inflammatory bowel disease (IBD)	1.4E−02	–
Pertussis	2.2E−05	–
Leishmaniasis	3.5E−02	–
Legionellosis	2.2E−02	–
Tuberculosis	3.9E−09	–
Amoebiasis	1.9E−03	–
Platelet activation	–	1.8E−03
Transcriptional misregulation in cancer	–	3.5E−04
Inflammatory mediator regulation of TRP channels	–	1.1E−03
Regulation of lipolysis in adipocytes	–	1.8E−02
Phosphatidylinositol signaling system	–	2.3E−04
Tight junction	–	1.8E−02
Inositol phosphate metabolism	–	1.5E−02
Axon guidance	–	7.7E−03
Notch signaling pathway	–	5.0E−04
PPAR signaling pathway	–	5.0E−02
Retinol metabolism	–	2.3E−02
Dilated cardiomyopathy	–	1.1E−02
Hypertrophic cardiomyopathy (HCM)	–	7.1E-03
Arrhythmogenic right ventricular cardiomyopathy (ARVC)	–	3.0E-04

FDRs of selected common pathways, unique human pathways, and unique mouse pathways are shown

## Discussion

We investigated the regulation characteristics of miR-132-3p in human and mouse by comparing murine and human validated and putative targets and exploring the pathways they are involved in. We found that human and murine share 6 validated targets, and that 9 additional transcripts contain a conserved MRE for miR-132-3p. Notably, the strict prediction algorithms used in this study predicted about 63% of the already validated targets in human, while the percent of shared predicted pathways was 87%. This could either reflect improved prediction power or a more profound conservation of the shared pathways in which this stress-controlling miRNA is involved.

Studying the regulatory impact and evolutionary conservation of miRNAs over stress responses presents challenging issues. While current humans carry an essentially similar genome to that of our ancient ancestors, the stressful experiences we are exposed to are largely different, and these differences are evidently larger in human-mouse comparisons. Therefore, we pursued an evolutionarily conserved miRNA that is known to be involved in stress reactions and where ample research had been done to identify its regulated targets and controlled pathways. In this context, miR-132 is especially suitable, as its interaction with AChE had been shown to control anxiety. Specifically, exposing mice to predator scent induces long-lasting hippocampal elevation of miR-132, accompanied by reduced AChE activity (Shaltiel et al. 2013; Zimmerman et al. 2012) as well as by epigenetic regulation via histone deacetylase 4 (Sailaja et al. 2012). Also, contextual fear conditioning increases pri-miR-132 levels in the hippocampus of chronically stressed rats (Meerson et al. 2010), as well as in the murine hippocampus (Ponomarev et al. 2011). Overall, these studies present miR-132′s involvement in overcoming stress-induced damage to protect cognitive function via its cholinergic control.

Surprisingly, the fraction of shared validated targets of miR-132-3p in the two species is less than a half, although miR-132 is fully conserved and 56–88% of the targets present a conserved MRE (validated and predicted MREs, respectively). One explanation could be that some of the targets are yet to be validated. In addition, context-dependent mechanisms at the cell, tissue, or organism level may potentially regulate miRNA-target interactions. Worth mentioning is the case of hsa-miR-132-3p and hsa-miR-212-3p that exhibit similar mature sequences and share the same seed region, yet only few targets were demonstrated to be targeted by both of them, and each of these miRNAs may also repress specific targets (Wanet et al. 2012). A related cell specific example was shown for miR-132 and SIRT1 interaction. Repressive effects of miR-132 on the 3′-UTR of SIRT1 were observed in HEK293T cells (Zhou et al. 2012) and in the liver (Hanin et al. 2017). In contrast, the SIRT1 3′-UTR failed to show similar effects in HepG2 cells, suggesting a context-specific regulation. Taking that into consideration, extending one context to others based on validation of targets in only one condition should be considered carefully.

We performed numerous tests in search for potential primate- and/or human-specific interactions of miR-132-3p. Searching for differences between the regulation of miR-132-3p in man and mice failed to identify any significant differences. We realize that the shared targets identified in our study are only part of the predicted ones in both species; nevertheless, the biological pathways controlled by these targets appear to be robustly shared. Furthermore, even when a predicted competition emerges with another miRNA, such as in the case of the MECP2 gene and miR-483, the spatial difference between the locations of the corresponding MREs makes such competition unlikely.

Another well-studied context of miR-132 is its activity in the immune system, known to be functionally involved in psychological stress responses (Mehta et al. 2015; Molnár et al. 2012; Taganov et al. 2006). MiR-132 is overexpressed in bacterial lipopolysaccharide (LPS)-stimulated primary human macrophages as well as in LPS-treated mice, where it attenuates inflammation by suppressing its stress-related target AChE (Shaked et al. 2009). This neuro-immune role (Soreq and Wolf 2011) elevates acetylcholine levels and induces blockade of NFkB-induced inflammation via the nicotinic acetylcholine receptor alpha 7 (Tracey 2010). At the transcript level, surface plasmon resonance analysis showed that miR-132 selectively targets the soluble splice variant of the acetylcholine hydrolyzing enzyme AChE-R (Mishra et al. 2017). Consequently, mice expressing an AChE transgene devoid of the miR-132 binding site develops high basal miR-132 expression levels, yet are incapable of controlling stress (Shaltiel et al. 2013) or inflammation (Shaked et al. 2009) via ACh. Taken together, these studies demonstrate inter-related stress/inflammation/neurodevelopment links for miR-132 but failed to identify any evolutionary modifications in any of those, excluding an option of primate and/or human-specific roles for this context. MiRNA regulation of stress responses thus emerges as being robustly preserved throughout mammalian species, primates included.

## Electronic Supplementary Material

Below is the link to the electronic supplementary material.

Supplementary material 1 (XLSX 31 kb)

Supplementary material 2 (XLSX 32 kb)

Supplementary material 3 (XLSX 12 kb)
